# Association of Vitamin D_3_ levels with glycemic control in Type 2 diabetes subjects from Gujarati population-India

**DOI:** 10.1186/1755-8166-7-S1-P36

**Published:** 2014-01-21

**Authors:** Avisek Majumder, Bhavik Doshi, Frenny Sheth, Manan Patel, Navneet Shah, Thakor Premal, Rama Vaidya, Jayesh Sheth

**Affiliations:** 1 FRIGE’s Institute of Human Genetics, FRIGE House, Jodhpur Gam Road, Satellite, Ahmedabad-380015, India; 2 Department of Diabetes and Endocrinology; Sterling Hospital, Ahmedabad- 380052, India; 3 Gujarat Diabetic Association, Ahmedabad-380007, India; 4 Kasturba Health Society, Medical Research Centre, Mumbai-400056, India

**Keywords:** Vitamin D_3_, HbA1C, Type 2 Diabetes

## Background

Considering the active role of Vitamin D_3_ in the functional regulation of pancreatic β-cells, present study was carried out to know the occurrence of Vitamin D_3_ deficiency in Type 2 diabetic [T2D] and non-diabetic subjects and demonstrate its influence on glycemic control.

## Materials and methods

This prospective study comprises of 508 individuals (including 210 T2D & 298 controls). All subjects were categorized into 3 groups according to Vitamin D_3_ levels. Group-I: included 171 individuals (61 T2D out of 171) with normal Vitamin D_3_ concentration (≥25 nmol/l), group-II: included 264 subjects (118 T2D out of 264)with mild to moderately Vitamin D_3_ deficiency (15-24.9 nmol/l) and group-III included 73 subjects (31 T2D out of 73) having severe Vitamin D_3_ deficiency (≤14.9 nmol/l). Vitamin D_3_ and glycosylated hemoglobin [HbA1C] level was analyzed for all the subjects.

## Results

Overall 66.34% subjects (both T2D & Controls) were found to have Vitamin D_3_ deficiency. This was more common in T2D patients (71%)(mean±SD Vitamin D_3_ level was 17.77±7.19nmol/L) as compared to controls (63%) (mean±SD Vitamin D3 level was 24.38 ± 9.30nmol/L)(p<0.05).Female subjects were more prone for Vitamin D_3_ deficiency as compared to male (71.09% vs 61.09%, p<0.03) subjects. Moreover, a gradual increase in mean HbA1C level was observed as Vitamin D_3_ level when reduced from its normal level in T2D subjects (7.95- 9.08% in group-I through group-III) [βHbA1C, Vit. D3= -0.07, r = -0.28, p<2.73×10^-5^]. No such changes in mean HbA1C level were observed in controls.

## Conclusion

Present study demonstrates the high prevalence (66.34%) of Vitamin D_3_ deficiency in Gujarati population from India, more so for subjects with T2D. It is likely that Vitamin D_3_ has a role in regulating insulin sensitization; resulting in poor glycemic control in subjects with low Vitamin D_3_ levels. This study also indicates that females are likely to be at a higher risk for Vitamin D_3_ deficiency compared to male in T2D and control subjects.

